# The Role of Information Entropy in Symmetry of Euclidean Polygons

**DOI:** 10.3390/e28050564

**Published:** 2026-05-18

**Authors:** Melvin M. Vopson

**Affiliations:** 1 School of Mathematics and Physics, University of Portsmouth, Portsmouth PO1 3QL, UK; melvin.vopson@port.ac.uk; 2 Information Physics Institute, Gosport PO12 3QP, UK

**Keywords:** information physics, information entropy, second law of infodynamics, euclidean polygons

## Abstract

In this paper we investigate the relationship between Shannon information entropy and symmetry in closed Euclidean polygons within the framework of the second law of information dynamics. Using Lagrange multiplier formalism, we derive the condition for minimum entropy in a system of fixed size, showing that it occurs when all elements have equal multiplicity. Applying this result to two-dimensional polygons, we demonstrate that zero-symmetry configurations maximize entropy, while maximally symmetric shapes correspond to minimum entropy states. We show that although entropy increases with geometric descriptor complexity for asymmetric shapes, it remains invariant for maximally symmetric configurations. These results provide a quantitative basis for the association between symmetry and low information entropy, within the broader framework of information dynamics and entropy minimization principles.

## 1. Introduction

Throughout history, the remarkable correspondence between mathematical equations and the behavior of the physical world has baffled scientists. The prevalence of mathematical equations, numbers, and symmetries that elegantly describe natural phenomena suggests a deeper connection between mathematics and the fundamental structure of the universe. Noether’s theorem establishes a link between symmetries and conservation laws [1]. Furthermore, the Standard Model of Particle Physics, which describes the fundamental particles and forces in the universe, is deeply rooted in mathematical symmetries [2]. Geometrical symmetries such as fractals, tessellations, and the Platonic solids are prevalent throughout the natural world. These symmetries mirror the symmetrical patterns that often arise in many biochemical processes, resulting in a wealth of biological structures that exhibit strong symmetries or regularity patterns [3]. The presence of such symmetries across different scales, from galaxies to subatomic particles, and biological life supports the idea that symmetry plays a pivotal role in the universe, connecting mathematics, chemistry, biology, and physics. Given that everything in the universe evolves to high entropy/maximum disorder, as dictated by the second law of thermodynamics, the widespread occurrence of symmetry in many natural systems is perplexing. Richard P. Feynman famously said [4]:

“*So our problem is to explain where symmetry comes from. Why is nature so nearly symmetrical? No one has any idea why.*”

Symmetry is a mathematical construct that describes the process in which a certain property is preserved when certain transformations are applied to a system or object. Symmetry transformations of a geometrical shape are called symmetry operations such as translations, rotations, reflections, and their combinations. Under such symmetry operations, an object remains invariant upon transformation. A symmetry operation is carried around a symmetry element, which can be a point, line, or plane about which a symmetry operation is carried out.

**Figure 1:**
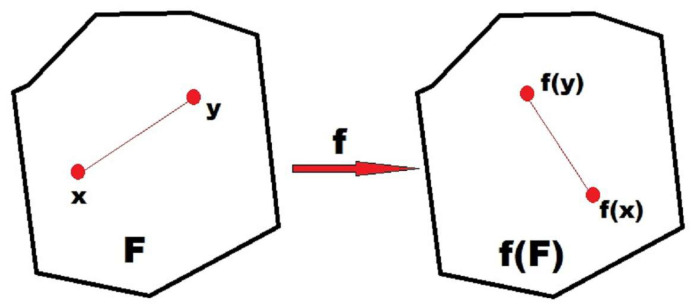
Schematic diagram of the 2D isometry mapping.

Mathematically, let us assume an n-dimensional (nD) geometry, where an nD figure is defined as any subset of the $R^{n}$ space. An isometry of the $R^{n}$ space is a function $f:R^{n}\to R^{n}$ that preserves distances, so for all $x_{1},x_{2},...x_{n}\in R^{n}$, the distance between any $f(x_{i})$ and $f(x_{j})$ is equal to the distance between $x_{i}$ and $x_{j}$. Therefore, mathematically, the symmetry of an nD figure F is an isometry mapping F into itself, so $f:R^{n}\to R^{n}$, such that f(F)=F. For a plane image, *n* = 2, so we have a 2D geometry (see diagram in Figure 1). The mathematical tool for the study of symmetry is the classical group theory, describing the structure of transformations that map objects to themselves exactly.

However, another interesting research direction concerned with the study of symmetries and their relationship to entropy and information has recently emerged. This is a promising field of research that can help to advance our understanding of the connections between mathematics, physics, biology, chemistry and information [5,6,7,8,9]. A number of studies reported the characterization of self-assembled 2D patterns and tessellations using the Voronoi and Shannon entropy [10,11,12]. The recently proposed second law of information dynamics proposes that information entropy in systems containing information may evolve toward reduced values at equilibrium, postulating that information entropy tends to a minimum value at equilibrium [13,14]. In fact, a link between the information entropy [15] and symmetries has been reported [14,16], demonstrating that the higher the symmetry, the lower the information entropy, and the maximal symmetry corresponds to the minimal information entropy content. This relationship suggests a possible information-theoretic interpretation for the widespread occurrence of symmetry in natural systems, consistent with the framework of the second law of information dynamics and information optimization principles. While previous studies have identified a qualitative relationship between symmetry and information entropy, indicating that highly symmetric systems correspond to lower entropy states [14,16], a general mathematical condition for the minimum attainable information entropy in systems of fixed size has not been explicitly derived.

In this work, we address this gap by formulating a constrained optimization problem for the Shannon entropy of a system with a fixed number of elements, and solving it using the Lagrange multipliers method. This approach yields a general and explicit condition for entropy minimization, namely that all elements must have equal multiplicity. We then apply this result to Euclidean polygons, demonstrating quantitatively how maximal symmetry corresponds to configurations of minimum information entropy. This provides a rigorous mathematical underpinning to the previously observed symmetry–entropy relationship in Euclidean polygons. The present analysis concerns Shannon information entropy associated with geometrical descriptors and should not be interpreted as a direct thermodynamic entropy calculation.

## 2. Information-Theoretic Framework

In Shannon’s information theory framework, let us consider a collection of *N* elements drawn from *n* distinct characters, so n≤N. The set of distinct characters $X=\left\{x_{1},x_{2},\ldots x_{n}\right\}$ has a discrete probability distribution on *X*, $P=\left\{p_{1},p_{2},\ldots p_{n}\right\}$. Hence, $p_{i}$ is the probability of $x_{i}$ to occur and the normalization is $\sum_{i=1}^{n}p_{i}=1$. The average information content per character is given by the Shannon information entropy formula

(1) $$I=-\sum_{i=1}^{n}p_{i}\cdot \log_{b}\left(p_{i}\right),$$

where the base of the logarithm, *b*, gives the units of information. When *b* = 2, the function returns an information value in bits. When *N = n*, the probability of each character $x_{i}$ to occur is $p_{i}=1/n=1/N$ for all $i=\bar{1,n}$. In this case the information content per character is maximum, $I=I_{max}=\log_{b}\left(N\right)$. This condition corresponds not only to the maximum information content, but also to the maximum thermodynamic disorder of the system in terms of microstates. When *n < N*, we have situations where some of the characters occur more than once. Let us define $g_{i}$ as the number of occurrences of $x_{i}$, or degeneracy of $x_{i}$. In this case, we can write the probabilities as $p_{i}=\frac{g_{i}}{N}$, so $P=\left\{\frac{g_{1}}{N},\frac{g_{2}}{N},\ldots \frac{g_{n}}{N}\right\}$. The information entropy is then:

(2) $$I=-\sum_{i=1}^{n}p_{i}\cdot \log_{b}\left(p_{i}\right)=\sum_{i=1}^{n}\frac{g_{i}}{N}\cdot \log_{b}\left(\frac{N}{g_{i}}\right)$$

The normalization condition $\sum_{i=1}^{n}p_{i}=1$ becomes:

(3) $$\sum_{i=1}^{n}g_{i}=N$$

Rearranging Equation (2) yields:

(4) $$I=\frac{log_{b}\left(N\right)}{N}\cdot \sum_{i=1}^{n}g_{i}-\frac{1}{N}\cdot \sum_{i=1}^{n}g_{i}\cdot log_{b}\left(g_{i}\right)$$

Using the normalization condition (3), relation (4) becomes:

(5) $$I=log_{b}\left(N\right)-\frac{1}{N}\cdot \sum_{i=1}^{n}g_{i}\cdot log_{b}\left(g_{i}\right)$$

Because the second term in relation (5) is always positive or zero, this relation shows that the information entropy has a maximum value, $I_{max}$, when the second term vanishes. This is the case when all $g_{i}=1$, so using (3) we get *n = N* and $I_{max}=log_{b}\left(N\right)=log_{b}\left(n\right)$. While the maximization of Shannon entropy for uniform probability distributions is a well-established result in information theory, the present analysis focuses specifically on the characterization of the minimum attainable information entropy under fixed system size *N*, formulated in terms of multiplicities $g_{i}$. The purpose of the present derivation is therefore not to establish a new entropy extremization principle, but rather to formulate the problem explicitly in terms of multiplicities $g_{i}$. This formulation is particularly suitable for applications involving geometrical symmetry in Euclidean polygons, where repeated geometrical descriptors naturally correspond to multiplicity structures, discussed in the following section. The question is:

*What is the minimum information entropy $I_{min}$ of a given system for the case n < N, and N constant?*

This question is relevant within the present information-theoretic framework because within the framework of the second law of information dynamics, the information entropy of a given system is interpreted as tending toward reduced values at equilibrium. Hence, instead of a general statement like this, the objective here is to derive the conditions required to obtain the minimum attainable information entropy of a system. Such relation may help identify equilibrium configurations of a given system, in addition to first-principle considerations. Examining (5), since $\frac{1}{N}\cdot \sum_{i=1}^{n}g_{i}\cdot log_{b}\left(g_{i}\right)>0$, then for a given *N* constant, the minimum *I* is obtained when the term $\frac{1}{N}\cdot \sum_{i=1}^{n}g_{i}\cdot log_{b}\left(g_{i}\right)$ is maximum. It is important to impose a constant *N* because the information entropy would naturally change due to changes in *N*. For instance, it has been shown that systems tend to reduce their information entropy indeed by reducing the overall information states, i.e., the number of *N* characters or events of the set *X*. Such case obviously leads to information entropy reduction, for instance, in digital data when self-erasure of bits occurs [13], or in genomic sequences when deletion mutations take place [14]. Here we are interested in the particular case when the evolution takes place while *N* remains constant, which is applicable to the calculation of the information entropy of geometrical shapes, for example. In order to determine the maximum/minimum value of $\frac{1}{N}\cdot \sum_{i=1}^{n}g_{i}\cdot log_{b}\left(g_{i}\right)$, we use the Lagrange multipliers method. Let us introduce a function

(6) $$f\left(g_{i}\right)=\frac{1}{N}\cdot \sum_{i=1}^{n}g_{i}\cdot log_{b}\left(g_{i}\right)$$

with the constraint $\sum_{i=1}^{n}g_{i}=N$, rewritten as

(7) $$q\left(g_{i}\right)=\sum_{i=1}^{n}g_{i}-N=0$$

The Lagrange multiplier function L is defined as

(8) $$L=f\left(g_{i}\right)-\lambda \cdot q\left(g_{i}\right)$$

where λ is called the Lagrange multiplier constant. The extreme is obtained when:

(9) $$\frac{\partial L}{\partial g_{i}}=\frac{\partial }{\partial g_{i}}\left(\frac{1}{N}\cdot \sum_{i=1}^{n}g_{i}\cdot log_{b}\left(g_{i}\right)-\lambda \cdot \left(\sum_{i=1}^{n}g_{i}-N\right)\right)=0$$

Taking *b* = 2 for binary units of information and solving (9) we obtain:

(10) $$log_{2}\left(g_{i}\right)+\frac{1}{ln(2)}=N\lambda$$

Changing the base of the logarithm from 2 to *e*, we get:

(11) $$log_{2}\left(g_{i}\right)=\frac{ln(g_{i})}{ln(2)}$$

Using (11) in (10) we obtain:

(12) $$g_{i}=\frac{2^{N\lambda }}{e}$$

Since all parameters in (12) are constants, $g_{i}=constant$, implying that the maximum or minimum of $f(g_{i})$ is obtained when all $g_{i}$ are equal: $g_{1}=g_{2}=\cdot \cdot \cdot =g_{n}=\frac{2^{N\lambda }}{e}$. Using this result together with the normalization condition (3), we obtain the Lagrange constant:

(13) $$\lambda =\frac{1}{N}\cdot log_{2}\left(\frac{eN}{n}\right)$$

Writing $log_{2}\left(e\right)=\frac{ln(e)}{ln(2)}=\frac{1}{ln(2)}$ in (10) and combining this with (13), we obtain:

(14) $$g_{i}=\frac{N}{n}$$

Condition (14) indicates that extremal information entropy states for fixed *N* occur when all $g_{i}$ are equal. When n≠1, the minimum information entropy is non-zero and the condition is given by (14). When n=1, so all *N* characters/events are identical, and the minimum information is $I_{min}=0$. When all $g_{i}$ are equal to 1, i.e., *n = N*, the information entropy takes the maximum possible value, $I_{max}=log_{2}\left(N\right)=log_{2}\left(n\right)$. This rule allows one to find the minimum attainable information entropy value at equilibrium for a system of *N* events, with n distinct events, when n≠1 and n<N.

## 3. Application to Euclidean Polygons

**Figure 2:**
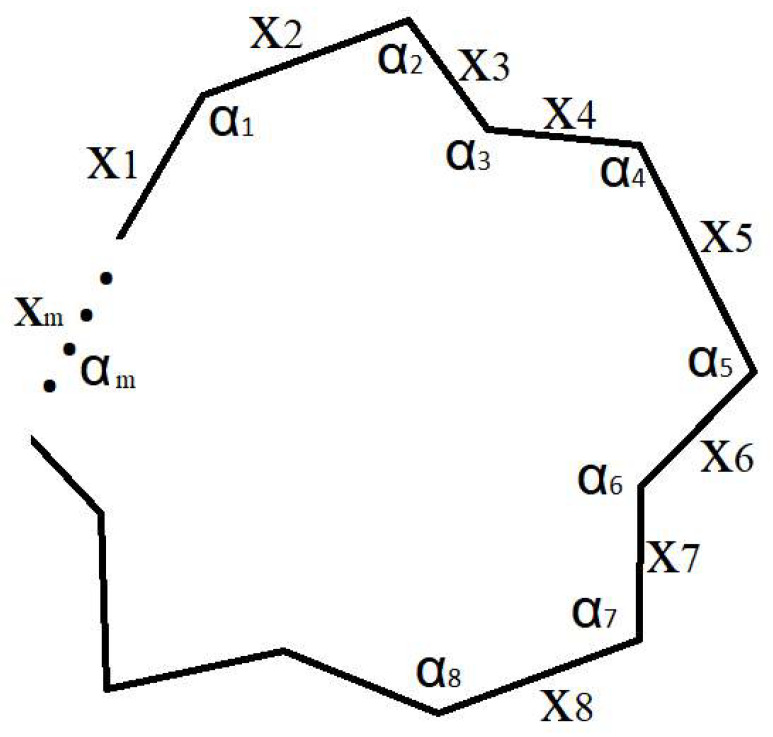
A random and irregular polygon consisting of *m* vertices, and with distinctive sides and angles.

In this work, a Euclidean polygon is defined as a closed two-dimensional geometrical figure embedded in Euclidean space $R^{2}$, bounded by a finite number of straight-line segments connected end-to-end. More generally, a Euclidean polygon refers to geometrical objects defined within flat Euclidean space satisfying the standard Euclidean axioms. In order to establish the relationship between the information entropy and symmetries, we need to define a way of quantifying how much symmetry a geometric shape has. This can be done by: (a) counting the number of symmetry operations possible; or (b) counting the number of symmetry elements. Using option (b), for instance, a perfect square has one axis of rotation and four axes of reflection, so we quantify its symmetry value as five, while a four-sided irregular polygon has no symmetry elements, so we quantify its symmetry as zero. The quantification of symmetry used here, based on counting symmetry elements, is adopted as a simple measure suitable for illustrative purposes. More generally, symmetry can be rigorously described using group theory, where the symmetry of a geometrical object is characterized by the structure and order of its symmetry group. In this framework, higher symmetry corresponds to a larger number of indistinguishable transformations, which is directly related to increased degeneracy in the description of the system. The present information-theoretic approach captures this degeneracy through the multiplicities $g_{i}$, providing a bridge between symmetry, group-theoretic invariance, and information entropy. Let us consider a closed random and irregular polygon consisting of *m* vertices with distinctive sides and angles (Figure 2).

**Figure 3:**
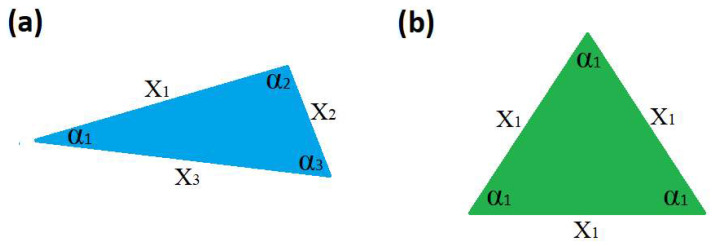
(**a**) A random triangle with no symmetry elements; (**b**) maximum symmetry—equilateral triangle.

The geometrical shape is fully described by knowing all *m* sides, $x_{i}$, and all *m* internal angles, $\alpha_{i}$. This also provides a measure of the geometric descriptor complexity, measured by the number of defining elements of a given polygon. These form a set *N* = 2*m* of characters containing *n = N* = 2*m* distinctive characters, so, $X=\left\{x_{1},x_{2},\ldots x_{m},\alpha_{1},\alpha_{2},\ldots ,\alpha_{m}\right\}$, having a discrete probability distribution on *X*, $P=\left\{p_{1},p_{2},\ldots p_{n}\right\}$. Since all $g_{i}$ are equal to 1, using (5), we determine that the information entropy of this polygon takes its maximum value, $I_{max}=log_{2}\left(N\right)=log_{2}\left(n\right)=log_{2}\left(2m\right)$. Any change to the image that would result in any $g_{i}$ becoming larger than 1 would result in a reduction of the information entropy, according to (5), while the minimum information entropy is obtained according to (14). In order to test this, let us consider a range of simple Euclidean polygon shapes. The smallest closed polygon is a triangle, and Figure 3a shows an ordinary triangle which has zero symmetry and Figure 3b shows an equilateral triangle that has the maximum symmetry.

For this shape, the set of characters is *N* = 2*m* = 6 and constant. The distinctive characters differ for the two shapes, so that *n* = *N* = 6 for the zero-symmetry shape, $X=\left\{x_{1},x_{2},x_{3},\alpha_{1},\alpha_{2},\alpha_{3}\right\}$, with a discrete probability distribution on *X*, $P=\left\{p_{1},p_{2},p_{3},p_{4},p_{5},p_{6}\right\}$. For the maximum symmetry equilateral triangle, we only have two distinct characters, *n* = 2, $X=\left\{x_{1},\alpha_{1}\right\}$. Relation (14) predicts the minimum information entropy for the case when all $g_{i}$ are equal and $g_{i}=N/n$.

**Figure 4:**
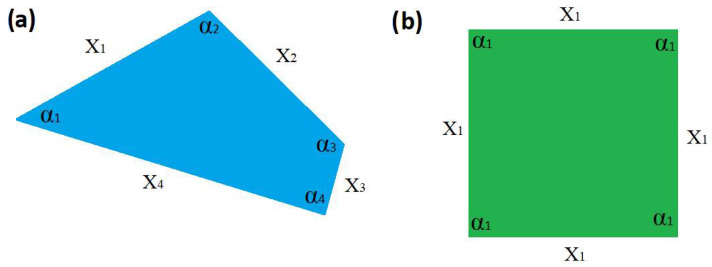
(**a**) A random quadrilateral shape with no symmetry elements; (**b**) maximum symmetry of a quadrilateral shape—square.

In our case, *N* = 6, *n* = 2, so this corresponds to $g_{i}=3$, which is exactly what we observe, so $P=\left\{p_{1},p_{2}\right\}=\left\{3/6,3/6\right\}$. Using (5), the information entropy for the two shapes is calculated as *I* = 2.585 bits for the ordinary triangle of zero symmetry and *I* = 1 bit for the equilateral triangle displaying maximum symmetry. Hence, we indeed observe that high symmetry corresponds to minimum information entropy. We now explore the applicability of the minimum information entropy rule to other geometric polygons of incremental size. Figure 4 shows the case of quadrilaterals, where Figure 4a has zero symmetry and Figure 4b displays the highest possible symmetry, i.e., a square. For this shape, the set of characters is *N* = 2*m* = 8. The zero symmetry is fully defined by eight distinctive characters, while the square has only two distinct characters, *n* = 2, $X=\left\{x_{1},\alpha_{1}\right\}$. According to (14), the minimum information entropy is achieved when $g_{i}=N/n$, which in this case corresponds to $g_{i}=4$. Again, this is exactly what we observe. Using (5), the information entropy for the two shapes is calculated as *I* = 3 bits for the ordinary quadrilateral of zero symmetry and *I* = 1 bits for the square.

**Figure 5:**
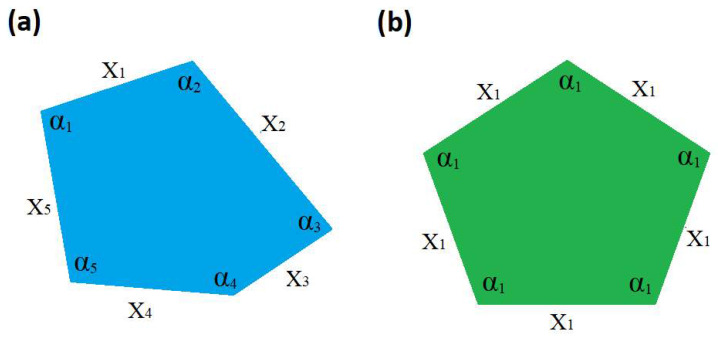
(**a**) An irregular pentagon with zero symmetry; (**b**) pentagon displaying maximum symmetry.

The minimal information entropy corresponding to the highest symmetry indeed checks for any Euclidean polygon shape. For the sake of clarity, here we demonstrate one more example of a five-sided polygon. Figure 5a shows the case of zero symmetry and Figure 5b shows the case of maximum symmetry, corresponding to a pentagon.

In this case, the set of characters is *N* = 10. The zero symmetry is fully defined by ten distinctive characters, while the pentagon with maximum symmetry has only two distinct characters, *n* = 2, $X=\left\{x_{1},\alpha_{1}\right\}$. The minimum information entropy corresponds to $g_{i}=5$, exactly matching the observation. The information entropy for the two five-sided polygons is calculated as *I* = 3.321 bits for the zero-symmetry shape and *I* = 1 bit for the pentagon.

**Figure 6:**
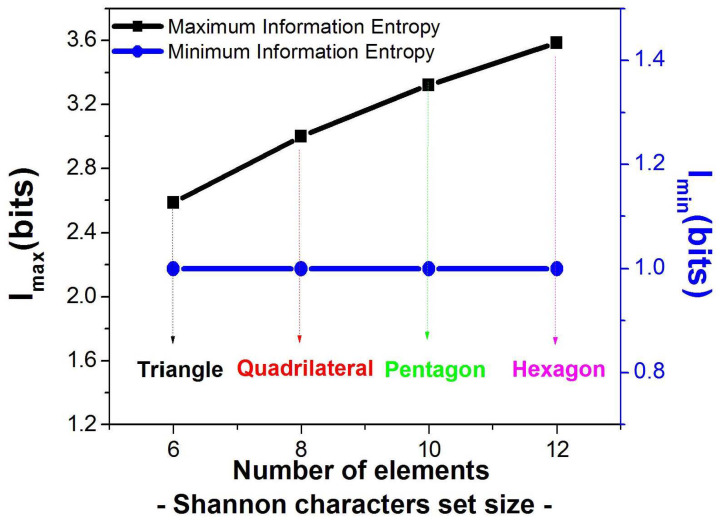
Maximum Information Entropy content of Euclidean polygons (left axis) with incremental geometric descriptor complexity corresponding to zero symmetry for each shape, and minimum Information entropy content for the same shapes in their maximal symmetry state (right axis).

An interesting observation is that for Euclidean polygons, the information content of zero-symmetry polygons increases linearly with the number of geometrical descriptors (sides and internal angles) required to define the shape, i.e., the information entropy increases. However, the information content of the highest-symmetry shapes is the same regardless of the geometric descriptor complexity (see Figure 6). This result demonstrates that the informational cost of asymmetric shapes increases with geometric descriptor complexity, whereas maximally symmetric configurations maintain constant minimal information content regardless of their geometric descriptor complexity (Figure 6).

## 4. Conclusions

In this study we investigated the relationship between Shannon information entropy and geometrical symmetry in Euclidean polygons within the framework of the second law of information dynamics. Building on prior work on the second law of infodynamics, suggesting that highly symmetric systems correspond to lower information entropy states, we develop a rigorous mathematical formulation to determine the conditions under which a system attains its minimum information entropy at a constant size. Using the method of Lagrange multipliers, we demonstrate that the minimum entropy configuration occurs when all elements describing the system have equal multiplicity, $g_{i}=N/n$. Applying this result to two-dimensional polygons, we show that irregular shapes with no symmetry exhibit maximal information entropy, whereas highly symmetric configurations correspond to minimal entropy states. Notably, while the information content of asymmetric shapes increases with geometric descriptor complexity, the entropy of maximally symmetric shapes remains constant regardless of geometric descriptor complexity. These findings provide a quantitative information-theoretic characterization of the relationship between geometrical symmetry and Shannon entropy in Euclidean polygons, indicating that highly symmetric configurations correspond to reduced information entropy states. Although the present analysis is restricted to static geometrical systems and does not address explicit physical dynamics, thermodynamic mechanisms, or temporal evolution, the results are consistent with broader information-theoretic interpretations linking symmetry, multiplicity, and entropy minimization principles. In this sense, the present work offers additional perspectives on the interplay between information theory and geometrical organization, and the results should be interpreted as an information-theoretic characterization of geometrical symmetry rather than a complete physical theory of symmetry formation. An interesting future extension of the present framework would involve the investigation of information entropy and symmetry relations in non-Euclidean geometries, including curved manifolds and topologically nontrivial spaces, as well as examining how information-theoretic symmetry measures evolve in dynamical systems, including the role of dissipation, self-organization, and symmetry emergence in physical and biological structures.

## Data Availability

All data used in this article is already available within the article.
